# Data from experiments with tailing material and Agrostis capillaris at three scales: pot, lysimeter and field plot

**DOI:** 10.1016/j.dib.2019.104964

**Published:** 2019-12-09

**Authors:** Aurora Neagoe, Paula Constantinescu, Andrei Nicoara, Marilena Onete, Virgil Iordache

**Affiliations:** a Research Centre for Ecological Services (CESEC), University of Bucharest, Aleea Portocalelor no. 1-3, 060101, Romania; b Bucharest Institute of Biology, Romanian Academy, Splaiul Independentei no. 296, Bucharest, Romania

**Keywords:** Tailings, Pot, Lysimeter, Field plot, Phytoremediaton, *Agrostis capillaris*

## Abstract

The data set consists in a file with two sheets: one includes a matrix of 297 rows and 46 columns, and the second one a matrix of 12 rows and 24 columns. In the first sheet each row is a replicate of an experimental variant with *Agrostis capillaris* growing on tailing substrate belonging to three experiments witch have the same variants, but are organized at three scales. The data from all experiments are in the same table, with a column indicating by a code the experiment to which they belong. In the second spreadsheet there is a table with the relative plant species cover in the experimental field plots. Experimental design and interpretation of the data are provided in “Implications of spatial heterogeneity of tailing material and time scale of vegetation growth processes for the design of phytostabilisation” [1].

**Table:** Specifications Table

Subject area	*Remediation of mining areas*
More specific subject area	*Phytoremediation of tailing dams with gentle techniques*
Type of data	*Table*
How data was acquired	*Hardware:* Microwave 3000 Anton Paar, flame AAS 5FL (Analytik Jena), centrifugal ball mill (MM400, grinding jar and ball zirconium oxide, Retsch), TOC Analyzer multi N/C 2100S (Co. Analytic, Jena, Germany), ICP-MS Perkin-Elmer ELAN DRC-e), CECIL Instruments Ltd. - Aquarius 190–1100 nm Double Beam Spectrophotometer *Methods:* as described in Ref. [1]
Data format	*Raw*
Experimental factors	One independent variable, type of amendment, with three nominal values: • tailing substate with 20% topsoil and 5% limestone (coded C) • C with 5% zeolite (coded CZ) • C with 5% zeolite and 5% raw *Trifolium* fertilizer (coded CZTr)
Experimental features	*Univariate pot, lysimeter and field plot experiment with type of amendment as independent variable. Five replicates of each variant for pots and lysimeters, four replicates for field plots.*
Data source location	*Zlatna, Romania, Valea Mica tailing dam, 46°09′32″N 23°13′16″E*
Data accessibility	*Data is with this article*
Related research article	*P. Constantinescu, A. Neagoe, A. Nicoară, A. Grawunder, S. Ion, M.Onete, V. Iordache, Implications of spatial heterogeneity of tailing material and time scale of vegetation growth processes on the design of phytostabilization, Sci. Total Environ., 692 (2019) 1057–1069* [1].

**Table:** 

**Value of the Data** • Data are useful because they are obtained in rarely implemented coupled experiments performed at three scales (pot, lysimeter and field plots) • Scientists in the fields of environmental biology, eco-physiology, plant science, agronomy, hazardous materials can benefit from this data. • Data can be used for further insights by metadata analysis. • Data about the plant variables can be integrate with data sets from unpolluted sites to explore the phenotypic plasticity of Agrostis capillaris. • Data can support the improvement of experimental design in order to obtain enough sample biomass at pot scale when the concentration of toxic elements in the tailing material are large, or to devise a better design of coupled multiple scales experiments. • The additional value of this data is related to the fact that Agrostis capillaris is our model plant species and other raw data sets about it will be reported in the future, corresponding to existing [2,3] and future publications. This will allow improve the potential for metadata analysis.

## 1 Data

The raw data reported here are in one Excel file with two spreadsheets. The first spreadsheet includes a matrix of 46 columns and 296 rows with all lab data resulting from sample analysis. Columns 1 to 4 in the first sheet include codes for experimental scale, experimental treatment, compartment (water, substrate, and plant parts) and sampling time. The other columns in the first sheet include data about the following measured variables: dry root biomass (raw and standardized), dry aboveground biomass (raw and standardized), proteins, superoxide dismutase activity, peroxidase activity, chlorophyll a and b, carotenes, lipids peroxidation in plants, soil respiration, electrical conductivity, pH, moisture, loss on ignition, cation exchange capacity, total carbon, N–NO^3-^, N–NO^2-^, N–NH^4+^,P-PO4^3-^ in amended substrate, Al, As, Ba, Be, Bi, Cd, Co, Cr, Cu, Li, Mg, Mn, Ni, P, Pb, Rb, Sr, U, V, Zn in tailing substrate and plants. In the second sheet each row is a replicate in the experiment performed at plot scale, in the field, and each column include data about the relative cover of a plant species.

## 2 Experimental design, materials, and methods

Full description of experimental design, materials, and methods is provided in Ref. [1] to which these data in brief are associated. No further information on how the data was created is available.
